# Inflammatory Biomarker Profiles in Odontogenic Infections: Evaluation of C-reactive Protein (CRP), Procalcitonin, WBC Count, and Neutrophil-to-Lymphocyte Ratio

**DOI:** 10.7759/cureus.106183

**Published:** 2026-03-31

**Authors:** C. Lalmuanpuia, Kartika Kaushik, Bagisha Kathuria, Alisha Angural, Sandip V Vasave, Shreya Chatterjee, Manish Sharma, Seema Gupta

**Affiliations:** 1 Department of Oral and Maxillofacial Surgery, Indira Gandhi Government Dental College and Hospital, Jammu, IND; 2 Department of Dental Surgery, Vardhman Mahavir Medical College, Safdarjung Hospital, New Delhi, IND; 3 Department of Oral Pathology, Government Dental College and Hospital, Jalgaon, IND; 4 Department of Laboratory Medicine, Rajendra Institute of Medical Sciences, Ranchi, IND; 5 Department of Oral Pathology, Jawahar Medical Foundation's Annasaheb Chudaman Patil Memorial Dental College, Dhule, IND; 6 Department of Orthodontics, Kothiwal Dental College and Research Centre, Moradabad, IND

**Keywords:** c-reactive protein, neutrophil-lymphocyte ratio, odontogenic infections, procalcitonin, white blood cells

## Abstract

Introduction: Odontogenic infections range from localized abscesses to severe, life-threatening conditions involving deep fascial spaces and systemic complications. Early identification of disease severity is essential for appropriate management and prevention of adverse outcomes. Inflammatory biomarkers, such as C-reactive protein (CRP), procalcitonin (PCT), white blood cell (WBC) count, and neutrophil-to-lymphocyte ratio (NLR), provide objective indicators of systemic infection and may aid in prognostic assessment. This study aimed to evaluate the levels of CRP, PCT, WBC count, and NLR in patients with odontogenic infections and determine their association with disease severity and clinical outcomes.

Materials and methods: This prospective cohort study included 50 patients with odontogenic infections who were admitted to the Jawahar Medical Foundation's AC Patil Memorial (ACPM) Dental College, Dhule, India, between January and December 2023. Severity was classified into four groups using a structured scoring system. Blood samples were collected upon admission to determine CRP, PCT, WBC count, and NLR levels. Statistical analysis was performed using the Kruskal-Wallis test, Spearman correlation, Mann-Whitney U test, regression analysis, and receiver operating characteristic (ROC) curve analysis.

Results: The mean age was 38.5 ± 14.2 years, with a male predominance. Median CRP was 72 mg/L, PCT 0.38 ng/mL, mean WBC 12.8 ± 3.8 ×10^3^/µL, and NLR 7.2 ± 4.3. All biomarkers increased significantly with disease severity (p < 0.001). PCT showed the strongest correlation with disease severity (ρ = 0.60), followed by NLR (ρ = 0.56). Patients with complications had significantly higher biomarker levels (p < 0.001). PCT demonstrated the highest predictive value for complications (odds ratio (OR) = 3.20). ROC analysis showed the highest accuracy for PCT (area under the curve (AUC) = 0.89), followed by NLR (0.86) and CRP (0.82).

Conclusion: Inflammatory biomarkers, particularly PCT and NLR, are reliable indicators of disease severity and predictors of clinical outcomes in odontogenic infections, supporting their use in early risk stratification and management.

## Introduction

Odontogenic infections, originating primarily from dental caries, periodontal disease, or periapical pathology, represent a significant proportion of head and neck infections encountered in clinical practice. These infections range from localized abscesses to severe, life-threatening conditions involving multiple fascial spaces, deep neck involvement, or systemic complications, such as sepsis, mediastinitis, or airway compromise [1]. Despite advances in antimicrobial therapy and surgical management, odontogenic infections remain a major cause of hospitalization, prolonged length of stay, intensive care admissions, and occasional mortality, particularly in vulnerable populations with comorbidities such as diabetes or immunosuppression [2]. The polymicrobial nature, often involving streptococci, anaerobes, and gram-negative rods, triggers a robust inflammatory response, leading to elevated systemic biomarkers that reflect infection severity and guide prognostic assessment [1].

Inflammatory biomarkers play a crucial role in the diagnosis, monitoring, and prediction of outcomes in infectious diseases, including odontogenic infections [3]. C-reactive protein (CRP), an acute-phase reactant synthesized by the liver in response to interleukin-6, rapidly increases within hours of infection onset, peaks at 24-48 hours, and declines with resolution, making it a sensitive indicator of inflammation severity, hospital length of stay, and treatment response [4]. White blood cell (WBC) count, particularly neutrophilia, indicates acute bacterial infection and host response, with elevated levels correlating with more extensive involvement or multiple-space infections [5]. Procalcitonin (PCT), a precursor to calcitonin produced in response to bacterial toxins, offers higher specificity for bacterial etiology than for viral causes and has shown utility in distinguishing localized from systemic infections, sepsis risk, and guiding antibiotic stewardship in maxillofacial contexts [6]. The neutrophil-to-lymphocyte ratio (NLR), derived from routine complete blood counts, serves as a cost-effective marker of systemic inflammation and stress; elevated NLR reflects neutrophil predominance and relative lymphopenia, associating with disease severity, prolonged hospitalization, antibiotic requirements, and complications, such as deep neck space infections or mortality [7].

Recent studies have highlighted the synergistic value of these biomarkers, such as combined CRP-NLR scores, in enhancing predictive accuracy for severe odontogenic infections, outperforming individual markers in receiver operating characteristic (ROC) analyses. Integrating traditional (CRP, WBC, PCT) and derived (NLR) parameters provides objective, measurable insights beyond clinical signs, aiding early risk stratification and management decisions [7,8]. The aim of this study was to evaluate the inflammatory biomarker profile in patients with odontogenic infections by assessing the levels of CRP, PCT, WBC count, and NLR, and to determine their association with infection severity and clinical outcomes. The objectives were: (1) to measure and compare serum levels of CRP, PCT, WBC count, and NLR in patients with odontogenic infections of varying severity; (2) to investigate correlations between these biomarkers and clinical parameters, such as extent of infection, length of hospital stay, need for intensive care, or complications; and (3) to assess the diagnostic and prognostic utility of individual and combined biomarkers in predicting severe disease progression.

## Materials and methods

This single-center prospective cohort study was conducted at the Jawahar Medical Foundation’s Annasaheb Chudaman Patil Memorial Dental College and Hospital, Dhule, Maharashtra, India, over the period from January 2023 to December 2023. The study adhered to the Strengthening the Reporting of Observational Studies in Epidemiology (STROBE) guidelines. Ethical approval (JMFACPMDC/IEC/2022/SS03) was obtained from the Institutional Ethics Committee, and written informed consent was obtained from all participants prior to enrolment.

Sample size estimation

We included 50 consecutive patients diagnosed with odontogenic infections and requiring hospitalization in this study. The sample size was calculated using G*Power software (version 3.1.9.7; Heinrich Heine University Düsseldorf, Düsseldorf, Germany) based on a large effect size (f = 0.6), α error of 0.05, and power of 80% for one-way analysis of variance (ANOVA) across four severity groups. The estimated sample size was 44, which was increased to 50 to account for potential dropouts and to enhance statistical robustness.

Inclusion and exclusion criteria

Patients aged ≥ 18 years presenting with acute odontogenic infections, such as abscesses, cellulitis, or fascial space involvement, with clinical features including swelling, fever (>38°C), leukocytosis, and radiographic confirmation, were included. Patients who had received antibiotics within the previous seven days were excluded. Additional exclusion criteria were non-odontogenic infections, uncontrolled systemic diseases (such as diabetes mellitus or immunosuppression), malignancy, pregnancy or lactation, chronic steroid therapy, prior maxillofacial trauma or surgery, and inability to provide informed consent.

Methodology

The severity of odontogenic infection was assessed using a structured severity scoring system adopted from a previous study that evaluates clinical parameters, including anatomical location, dysphagia, aphagia, dyspnea, fever, sepsis, and necrotic stage [9]. Based on these criteria, patients were categorized into four severity levels: low, moderate, high, and extremely high (Table 1).

**Table 1:** Infection severity score was obtained according to scale given in a previous study [9]. Source: Reproduced from [9] under a Creative Commons license.

Point	Severity	Description
1	Low	One or more criterion with lower severity, except dysphagia + dyspnoea
2	Moderate	One criterion with moderate severity, or Low severity of dysphagia + Low severity of dyspnoea
3	High	One criterion with high severity, or at least two criteria with moderate severity
4	Extremely high	One criterion with extremely high severity, or at least two criteria with high severity, or Aphagia, or Sepsis, or Necrotic stage

Venous blood samples (5 mL) were collected from each patient upon admission under aseptic conditions. Samples were centrifuged at 3000 rpm for 10 min, and the serum was either analyzed immediately or stored at -80^°^C until further use. CRP levels were measured using an immunoturbidimetric assay, whereas PCT levels were determined using a chemiluminescent immunoassay. Complete blood counts were performed using an automated hematology analyzer, and the NLR was calculated by dividing the absolute neutrophil count by the lymphocyte count. All laboratory analyses were conducted in an accredited diagnostic laboratory, with duplicate measurements performed to ensure quality control.

Data analysis

The primary outcome measures included infection severity score, length of hospital stay, development of complications, and treatment response. Statistical analyses were performed using the IBM SPSS Statistics for Windows, Version 26 (Released 2018; IBM Corp., Armonk, New York, United States). Data normality was assessed using the Shapiro-Wilk test, which revealed a non-normal distribution for most variables. Accordingly, non-parametric tests were employed. Differences in biomarker levels across severity groups were analyzed using the Kruskal-Wallis test with a post hoc Bonferroni correction. Correlations between biomarkers and severity scores were evaluated using Spearman’s rank correlation coefficient. The Mann-Whitney U test was used to compare patients with and without complications. ROC curve analysis was performed to determine optimal cutoff values, sensitivity, specificity, and area under the curve (AUC). Univariate logistic regression analysis was conducted to identify predictors of complications, whereas linear regression analysis was used to assess predictors of hospital length of stay. Statistical significance was set at P < 0.05.

## Results

We included 50 patients diagnosed with odontogenic infections in this study. The mean age of the study population was 38.5 ± 14.2 years, with a male predominance. Baseline inflammatory biomarkers were elevated, indicating an active infection. The median CRP level was 72 mg/L (interquartile range, 25-165 mg/L), and the median PCT level was 0.38 ng/mL (interquartile range, 0.12-1.20 ng/mL). The mean WBC count was 12.8 ± 3.8 ×10^3^/µL, and the mean NLR was 7.2 ± 4.3 (Table 2).

**Table 2:** Baseline characteristics of the study sample. Values are presented as mean ± standard deviation (SD) or median (interquartile range, IQR) as appropriate. CRP: C-reactive protein, WBC: white blood cell count, NLR: neutrophil-to-lymphocyte ratio

Variable	Value
Age (years)	38.5 ± 14.2
Sex (M/F)	30 (60%)/20 (40%)
CRP (mg/L)	72 (25-165)
Procalcitonin (ng/mL)	0.38 (0.12-1.20)
WBC count (×10^3^/µL)	12.8 ± 3.8
NLR	7.2 ± 4.3

A progressive and statistically significant increase in all inflammatory biomarkers was observed across the four severity groups (low, moderate, high, and extremely high). CRP levels increased from 30.50 ± 12.40 mg/L in the low-severity group to 198.60 ± 60.10 mg/L in the extremely high-severity group. Similarly, PCT levels increased from 0.15 ± 0.08 ng/mL to 1.65 ± 0.70 ng/mL across the severity spectrum. WBC count increased from 9.80 ± 2.10 ×10^3^/µL to 17.20 ± 3.80 ×10^3^/µL, while NLR increased from 3.50 ± 1.50 to 13.50 ± 4.20. These differences were statistically significant (p < 0.001 for CRP, PCT, and NLR; p = 0.001 for WBC), demonstrating a strong association between biomarker levels and infection severity (Table 3).

**Table 3:** Comparison of inflammatory biomarkers across severity groups. Values are expressed as mean ± SD. Statistical analysis was performed using the Kruskal-Wallis test. * Statistically significant at p < 0.05. CRP: C-reactive protein, WBC: white blood cell count, NLR: neutrophil-to-lymphocyte ratio

Biomarker	Low (n = 8)	Moderate (n = 22)	High (n = 15)	Extreme (n = 5)	H-value	p-value
CRP (mg/L)	30.50 ± 12.40	72.30 ± 28.60	130.50 ± 45.20	198.60 ± 60.10	18.60	<0.001*
Procalcitonin (ng/mL)	0.15 ± 0.08	0.38 ± 0.20	0.85 ± 0.35	1.65 ± 0.70	16.90	<0.001*
WBC (×10^3^/µL)	9.80 ± 2.10	12.10 ± 2.80	14.50 ± 3.20	17.20 ± 3.80	10.80	0.001*
NLR	3.50 ± 1.50	6.20 ± 2.40	9.80 ± 3.50	13.50 ± 4.20	15.40	<0.001*

Correlation analysis revealed significant positive relationships between all biomarkers and severity scores. PCT demonstrated the strongest correlation (ρ = 0.60, p < 0.001), followed by NLR (ρ = 0.56, p < 0.001) and CRP (ρ = 0.52, p < 0.001). WBC count showed a comparatively weaker but still significant correlation (ρ = 0.41, p = 0.003). These findings indicate that PCT and NLR are more reliable indicators of disease severity than traditional markers (Table 4).

**Table 4:** Correlation between biomarkers and severity score. Spearman’s rank correlation coefficient (ρ) was used to assess association. *Statistically significant at p < 0.05. CRP: C-reactive protein, WBC: white blood cell count, NLR: neutrophil-to-lymphocyte ratio

Biomarker	Correlation coefficient (ρ)	p-value
CRP vs. Severity	0.52	<0.001*
Procalcitonin vs. Severity	0.60	<0.001*
WBC vs. Severity	0.41	0.003*
NLR vs. Severity	0.56	<0.001*

Of the 50 patients, 10 developed complications during the course of treatment. Patients with complications exhibited significantly higher biomarker levels than those without complications. The mean CRP levels were 165.20 ± 55.30 mg/L in the complication group compared with 68.40 ± 35.20 mg/L in the non-complication group. PCT levels were markedly elevated (1.35 ± 0.65 ng/mL vs. 0.38 ± 0.22 ng/mL), while NLR and WBC counts were also significantly higher in patients with complications. These differences were statistically significant (p < 0.001 for CRP, PCT, and NLR; p = 0.002 for WBC), indicating their role in identifying high-risk patients (Table 5).

**Table 5:** Comparison of biomarkers between patients with and without complications. Values are expressed as mean ± SD. The Mann-Whitney U test was used for comparison. *Statistically significant at p < 0.05. CRP: C-reactive protein, WBC: white blood cell count, NLR: neutrophil-to-lymphocyte ratio

Biomarker	Complications (n = 10)	No complications (n = 40)	p-value
CRP (mg/L)	165.20 ± 55.30	68.40 ± 35.20	<0.001*
Procalcitonin (ng/mL)	1.350 ± 0.65	0.38 ± 0.22	<0.001*
WBC (×10^3^/µL)	15.80 ± 3.50	12.00 ± 3.20	0.002*
NLR	12.80 ± 4.20	6.50 ± 3.10	<0.001*

Univariate logistic regression analysis demonstrated that all biomarkers were significant predictors of complications. PCT showed the highest predictive value (odds ratio (OR): 3.20, 95% CI: 1.60-6.80, p = 0.002), followed by NLR (OR: 1.30, p = 0.003), CRP (OR: 1.18 per 10 mg/L increase, p = 0.004), and WBC count (OR: 1.22, p = 0.01). These findings confirmed that elevated admission biomarker levels significantly increased the likelihood of developing complications (Table 6).

**Table 6:** Univariate logistic regression analysis for predictors of complications. Logistic regression analysis was performed to identify predictors of complications. *Statistically significant at p < 0.05. CRP: C-reactive protein, WBC: white blood cell count, NLR: neutrophil-to-lymphocyte ratio

Variable	Odds ratio (OR)	95% CI	p-value
CRP (per 10 mg/L increase)	1.18	1.05-1.32	0.004*
Procalcitonin	3.20	1.60-6.80	0.002*
WBC	1.22	1.05-1.45	0.01*
NLR	1.30	1.10-1.60	0.003*

Linear regression analysis revealed that all biomarkers were significantly associated with an increased length of hospital stay. NLR demonstrated the strongest association (β = 0.28, p = 0.004), followed by PCT (β = 1.25, p = 0.01). The CRP level (β = 0.015, p = 0.01) and WBC count (β = 0.22, p = 0.03) also showed significant positive associations. These results indicate that higher biomarker levels at admission are predictive of prolonged hospitalization (Table 7).

**Table 7:** Linear regression analysis for predictors of length of hospital stay. Linear regression analysis was performed to evaluate predictors of hospital length of stay. *Statistically significant at p < 0.05. CRP: C-reactive protein, WBC: white blood cell count, NLR: neutrophil-to-lymphocyte ratio

Variable	Beta coefficient	Standard error	p-value
CRP	0.015	0.006	0.01*
Procalcitonin	1.25	0.50	0.01*
WBC	0.22	0.10	0.03*
NLR	0.28	0.09	0.004*

**Figure 1:**
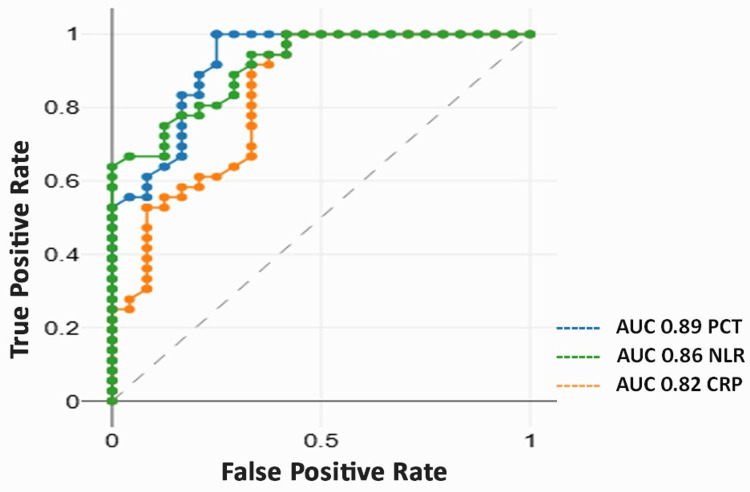
Receiver operating characteristic (ROC) curve analysis of inflammatory biomarkers for predicting severe odontogenic infections. PCT: procalcitonin; CRP: C-reactive protein; NLR: neutrophil-to-lymphocyte ratio; AUC: area under the curve

ROC curve analysis demonstrated excellent diagnostic performance of the biomarkers in predicting severe outcomes. PCT exhibited the highest accuracy with an AUC of 0.89 (95% confidence interval (CI): 0.78-0.97, p < 0.001), followed by NLR (AUC = 0.86) and CRP (AUC = 0.82). The optimal cutoff values were identified as 0.48 ng/mL for PCT (85% sensitivity, 82% specificity), 9.0 for NLR (82% sensitivity, 78% specificity), and 135 mg/L for CRP (78% sensitivity, 75% specificity). These findings highlight the strong diagnostic and prognostic utility of these biomarkers in odontogenic infections (Table 8 and Figure 1).

**Table 8:** Receiver operating characteristic (ROC) curve analysis for predicting severe outcomes. ROC analysis was performed to determine diagnostic performance. *Statistically significant at p < 0.05. CRP: C-reactive protein, NLR: neutrophil-to-lymphocyte ratio; AUC: area under the curve, CI: confidence interval

Biomarker	AUC	95% CI	Cutoff	Sensitivity (%)	Specificity (%)	p-value
CRP	0.82	0.70-0.94	135.00 mg/L	78	75	<0.001*
Procalcitonin	0.89	0.78-0.97	0.48 ng/mL	85	82	<0.001*
NLR	0.86	0.75-0.95	9.00	82	78	<0.001*

## Discussion

The present study evaluated the roles of inflammatory biomarkers, including CRP, PCT, WBC count, and NLR, in assessing the severity and clinical outcomes of odontogenic infections. The findings demonstrated that all biomarkers were significantly associated with disease severity, complications, and length of hospital stay, highlighting their potential utility in clinical decision-making and prognostic stratification.

A key observation of this study was the progressive elevation of all biomarkers with increasing severity of odontogenic infections. This trend reflects the underlying pathophysiology of infection, in which bacterial invasion triggers a systemic inflammatory response mediated by cytokines, such as interleukin-6 and tumor necrosis factor-alpha. CRP, an acute-phase reactant synthesized in the liver, increases rapidly in response to inflammation, whereas WBC count reflects leukocyte mobilization against infection. Similarly, NLR serves as a composite marker of systemic stress, indicating neutrophilia and relative lymphopenia, both of which are characteristic of acute bacterial infections. These findings are consistent with previous studies that have demonstrated a strong association between elevated inflammatory markers and the severity of odontogenic and deep neck space infections [4,10,11].

Among the evaluated biomarkers, PCT showed the strongest correlation with infection severity and complications. This finding can be attributed to its higher specificity for bacterial infections compared to other markers [6,12]. PCT is released in response to bacterial endotoxins and systemic inflammatory mediators, and its levels increase proportionally with infection severity and the risk of sepsis [12]. Previous studies have reported similar findings, indicating that PCT is a reliable marker for distinguishing localized infections from systemic involvement and for predicting severe outcomes [6,12,13]. Its superior diagnostic accuracy in the present study further supports its role as a valuable biomarker for maxillofacial infections.

The NLR was strongly associated with disease severity and clinical outcomes. As a readily available and cost-effective parameter derived from routine blood tests, the NLR reflects the balance between innate and adaptive immune responses. Elevated NLR values indicate increased neutrophilic activity and suppressed lymphocyte-mediated immunity, both of which are indicative of severe systemic inflammation [14]. These findings align with previous research that has identified the NLR as an independent predictor of severity, complications, and prolonged hospitalization in odontogenic and other infectious conditions [7,15]. The strong predictive performance of the NLR observed in this study reinforces its clinical utility as an accessible biomarker.

Although WBC count was significantly associated with severity and complications, its predictive value was comparatively lower than that of PCT and NLR. This may be due to the nonspecific nature of leukocytosis, which can occur in a variety of inflammatory and physiological conditions. Similar observations have been reported in previous studies, wherein WBC count alone was found to have limited specificity in assessing infection severity [10,16]. Therefore, reliance on WBC count as a sole indicator may be inadequate, and its use should be complemented with more specific biomarkers.

The association between elevated biomarker levels and increased risk of complications highlights the importance of early identification of high-risk patients. Patients with higher biomarker levels are more likely to develop complications and require prolonged hospitalization, suggesting that these markers can aid in early risk stratification and resource allocation. Early identification of severe cases allows for timely intervention, including aggressive surgical management, appropriate antibiotic therapy, and close monitoring, thereby improving patient outcomes. These findings are in agreement with those of previous studies that have demonstrated the prognostic value of inflammatory markers in predicting adverse outcomes in head and neck infections [3-7].

Clinical implications and limitations

The findings of this study have several important clinical implications. The use of inflammatory biomarkers, particularly PCT and NLR, can enhance early diagnosis, severity assessment, and prognostic evaluation of patients with odontogenic infections. Incorporating these biomarkers into routine clinical practice may facilitate timely decision-making, optimize treatment strategies, and reduce the risk of complications. Additionally, the application of a structured severity scoring system provides a standardized approach for patient stratification, which can improve communication among clinicians and guide management protocols.

However, certain limitations should be acknowledged. The study was conducted at a single center with a relatively small sample size, which may limit the generalizability of the findings. The absence of a multivariate regression analysis restricts the ability to determine independent predictors while controlling for confounding variables. Furthermore, serial measurements of biomarkers were not extensively analyzed, which could have provided additional insights into disease progression and treatment response. Future multicenter studies with larger sample sizes and longitudinal biomarker assessments are recommended to validate and expand upon these findings.

## Conclusions

Inflammatory biomarkers, particularly PCT and NLR, have demonstrated strong diagnostic and prognostic value in assessing the severity of odontogenic infections. Their significant association with disease severity, complications, and length of hospital stay highlights their utility in early risk stratification and clinical decision-making. While traditional markers such as CRP and WBC remain useful, PCT has shown superior predictive performance. The application of a structured severity scoring system further enhances the objective assessment. Incorporating these biomarkers into routine clinical practice may improve patient outcomes through timely intervention, optimized management strategies, and better identification of high-risk cases requiring intensive care.
